# Coral restoration can drive rapid increases in reef accretion potential

**DOI:** 10.1038/s41598-025-04818-3

**Published:** 2025-08-04

**Authors:** Lauren T. Toth, Selena A. Johnson, Erin O. Lyons, Jason Spadaro, Anastasios Stathakopoulos, Sierra Bloomer, Jennifer Mallon, Connor M. Jenkins, Sara D. Williams, Ian Combs, Zachary Craig, Erinn Muller

**Affiliations:** 1 St. Petersburg Coastal and Marine Science Center, U.S. Geological Survey, St. Petersburg, FL USA; 2 Cherokee Nations System Solutions, Tulsa, OK USA; 3 Mote Marine Laboratory, Summerland Key, FL USA; 4 National Coral Reef Institute, Halmos College of Arts and Sciences, Nova Southeastern University, Dania Beach, FL USA; 5 Mote Marine Laboratory, Sarasota, FL USA; 6 Division of Aquatic Resources, Hawai’i Department of Land and National Resources, Kailua-Kona, HI USA

**Keywords:** Restoration ecology, Marine biology, Ecosystem ecology, Climate change

## Abstract

Coral-reef degradation is disrupting the balance between reef accretion and erosion and threatening the persistence of essential coral-reef habitats. In south Florida, most reefs are already net eroding, and without intervention, valuable ecosystem services may be lost. Coral restoration holds the potential to reverse those trends; however, typical restoration monitoring does not adequately capture key geo-ecological functions. We addressed this knowledge gap using carbonate budgets and Structure-from-Motion models to evaluate the impact of coral restoration on reef-accretion potential and structural complexity at eight offshore and three inshore coral reefs in the Lower Florida Keys. Within 2–6 years following outplanting, restoration of rapidly growing *A. cervicornis* populations increased reef-accretion potential to 2.8 mm y^− 1^ and drove significant increases in structural complexity. There was no measurable impact of restoring slower-growing, massive corals on reef-accretion potential inshore; however, whereas the severe 2023 coral-bleaching event immediately following our study caused near-complete mortality of *A. cervicornis*, 59% of massive corals survived, highlighting potential trade-offs between coral growth and survival on future restoration efficacy. We conclude that although restoration can produce rapid, small-scale increases in reef-accretion potential, there remain important uncertainties about how and whether ecosystem-scale benefits of restoration on important geo-ecological reef functions can persist long term.

**Supplementary Information:**

The online version contains supplementary material available at 10.1038/s41598-025-04818-3.

## Introduction

Coral reefs create essential habitat that supports biodiversity, fisheries production, tourism, and shoreline protection: ecosystem services that are valued at more than $8.5 billion in south Florida alone^1^. The persistence of reef habitats, and the critical ecological and socioeconomic functions they support, relies on the balance between the processes of reef growth (or “accretion”) and erosion^2,3^. For thousands of years, growth of corals and other calcifying organisms has been sufficient for most reefs to overcome background rates of physical, chemical, and biological erosion, allowing for the accretion of the complex three-dimensional reef structures present today^4–7^; however, the unprecedented mortality of reef-building corals over the last half a century as a result of thermal stress, disease, and other local-scale stressors is increasingly disrupting that equilibrium^3,8–11^. On the subtropical reefs of south Florida, where the suboptimal climatic setting has limited reef development for millennia^6,12^, the impacts of recent coral mortality have been particularly extreme^13–18^. Erosion is now the dominant process on most of Florida’s coral reefs^16,19^ and many reef structures that took thousands of years to build are now eroding away^20,21^.

Because the geological architecture of reefs serves as the structural foundation for the myriad ecosystem services reefs provide to society, reviving the reef-accretion process is the most fundamental challenge in coral-reef management^3,22,23^. It is also a challenge that coral restoration is uniquely poised to address; by enhancing the cover of living corals, coral restoration not only directly increases structural complexity and reef-accretion capacity^24–27^ it can also reduce the amount of exposed reef area that is vulnerable to erosion^2,28^. Although preservation of key geo-ecological functions, such as reef accretion, have increasingly been cited as important goals of coral restoration programs^29,30^, limited time and resources for monitoring following outplanting efforts have often made it infeasible for practitioners to evaluate functional impacts^31–33^.

Carbonate-budget models can provide a powerful tool for evaluating restoration success that goes beyond the typical measures of coral health or survival. Carbonate budgets are constructed by conducting a census of the calcifying and bioeroding taxa on a reef to estimate the balance between reef accretion and erosion^34,35^. Although carbonate-budget models do not include the important impacts of chemical dissolution and physical erosion on net reef accretion^36–39^ they nonetheless provide a powerful means of quantifying and comparing spatial and temporal differences in reef-accretion potential^8,10,40^. Carbonate-budget modeling has also recently been used to demonstrate how planned coral restoration could improve the reef-accretion process in the coming decades^16^ (e.g., based on the Mission: Iconic Reefs plan^41^), to evaluate how projections of climate change could impact restoration efficacy^42^ and to quantify the magnitude of restoration effort required to mitigate the worst impacts of sea-level rise and hurricanes on coastal-flooding hazards by the end of the century^43^.

**Fig. 1:**
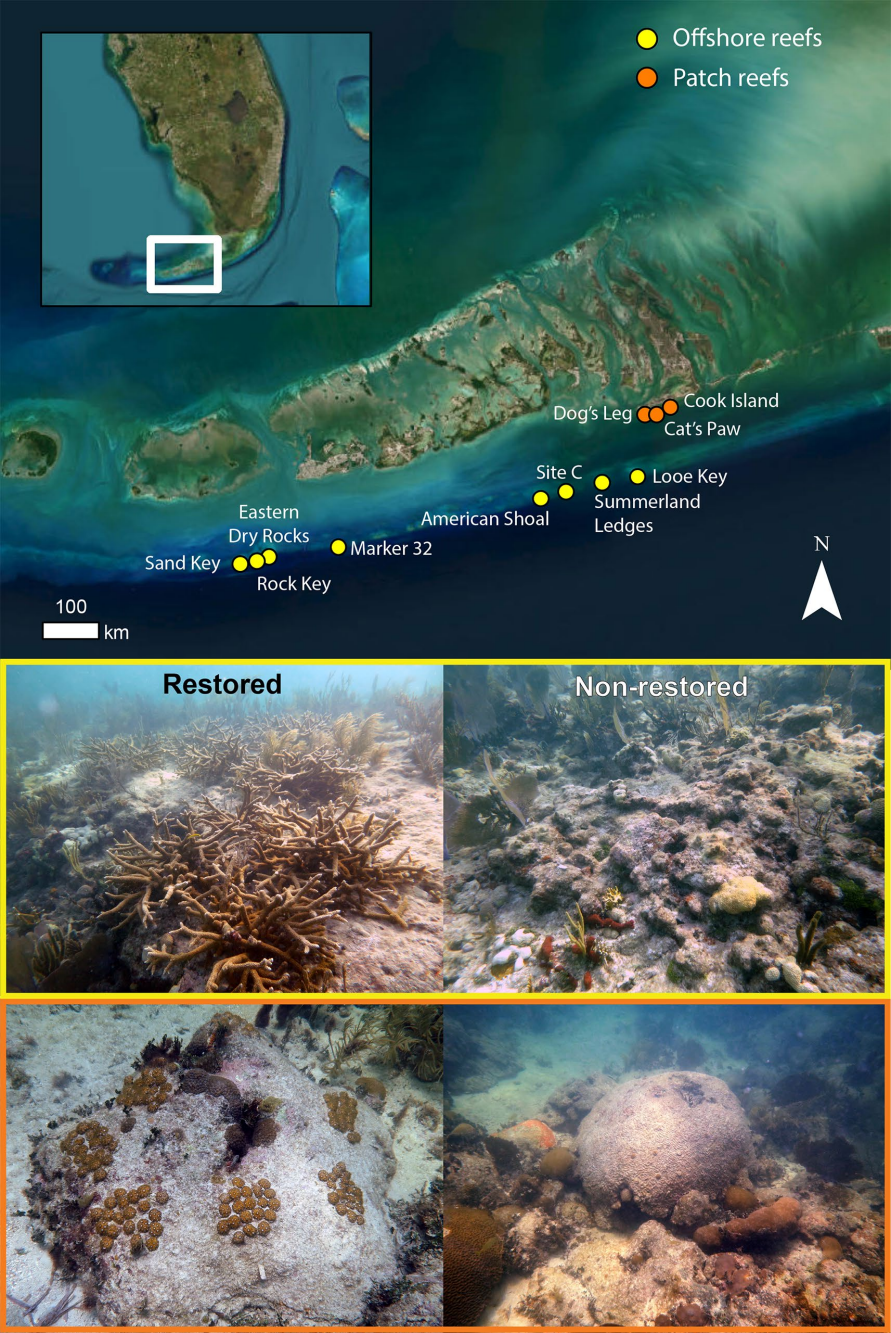
Location of reefs surveyed in this study. Map of study locations (upper panel) with examples of restored (left) and non-restored (right) areas of the offshore (yellow; American Shoal) and patch-reef sites (orange; Cook Island). Photos were taken by L.T. Toth and E.O. Lyons in July 2022. The map was generated by L.T. Toth using the software ArcGIS Pro version 3.5. Map image is the intellectual property of Esri and is used herein under license. Copyright 2024 Esri and its licensors. All rights reserved.

Quantifying how and whether coral restoration could reverse declines in reef-accretion potential is essential to demonstrating the broader functional and socioeconomic impacts of coral-restoration programs. Although there has been important recent progress in demonstrating the functional impact of coral restoration *in theory*, only a handful of studies have quantified how restoration is affecting the reef-accretion process *in practice*^25–27,45^. We address this critical knowledge gap by comparing coral-reef carbonate budgets and structural complexity at outplanted, or “restored”, and non-restored areas of eight offshore fore-reef and three inshore patch-reef sites in the Lower Florida Keys where Mote Marine Laboratory researchers have been outplanting corals since 2016 (Fig. 1; Table 1; see Methods). We explore the potential efficacy of restoration for reviving reef ecosystem function and discuss trade-offs among different restoration strategies in the context of the unprecedented 2023 coral-bleaching event.

**Table 1:** Summary of reefs surveyed in this study.

Description of restoration	Site name (# subsites)	Latitude	Longitude	Outplanting years	Survey year (# years of restoration)	~Outplants per subsite
Offshore reef with *Acropora cervicornis* outplanting	Sand Key (4)	24.452	− 81.876	2018–2021	2023 (2–5)	500–1000
	Rock Key (2)	24.455	− 81.859	2016	2023 (6)	1000
	Eastern Dry Rocks (3)	24.459	− 81.844	2016–2020	2023 (3–6)	500–1000
	Marker 32 (2)	24.474	− 81.743	2017 & 2019	2023 (4–6)	500–600
	American Shoal (2)	24.523	− 81.519	2018 & 2019	2022 (3–4)	500–550
	Mote site C (3)	24.531	− 81.488	2019	2022 (3)	500
	Summerland Ledges (3)	24.540	− 81.445	2020 & 2021	2022 (1–2)	250
	Looe Key (2)	24.546	− 81.403	2018 & 2019	2022 (3–4)	550–800
Inshore patch reef with massive coral outplanting	Dog’s Leg (2)	24.615	− 81.384	2020–2022	2023 (1–3)	315–500
	Cat’s Paw (2)	24.615	− 81.380	2019 & 2020	2023 (3–4)	220–345
	Cook Island (1)	24.622	− 81.363	2011–2017	2023 (6–12)	870

Descriptions of restoration activities, timing, and effort at restoration locations (subsites) within each reef. The number of outplanted corals at each subsite are approximate (~). Additional site details, including names of subsites, are available in a U.S. Geological survey data release^44^.

## Results

### Carbonate budgets

**Fig. 2:**
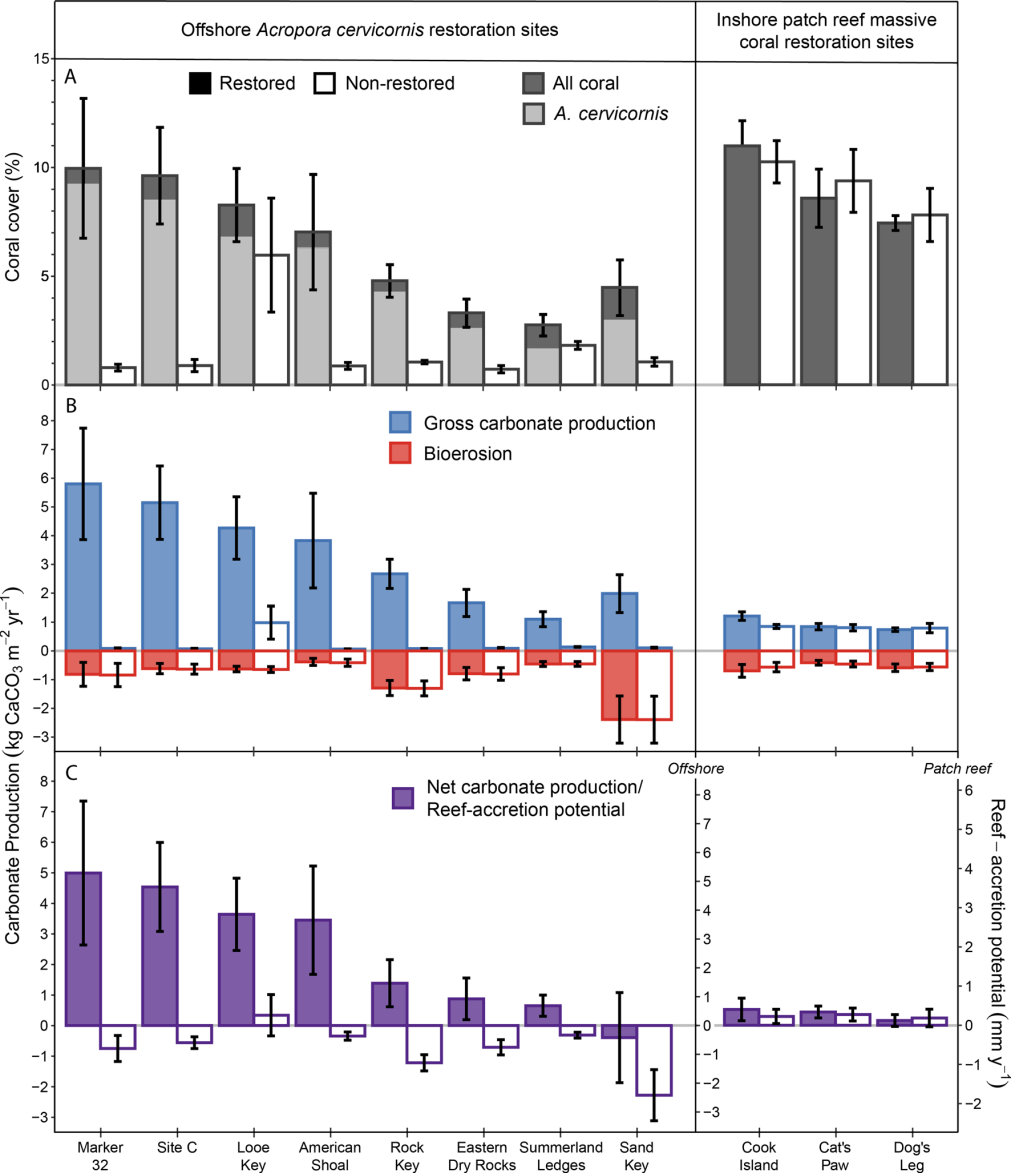
The impact of restoration on coral-reef carbonate budgets. Differences in mean (**A**) coral cover, (**B**) gross carbonate production and bioerosion, and (**C**) net carbonate production (left axis) and reef-accretion potential (right axis) between restored (filled bars) and non-restored (open bars) areas of offshore *Acropora cervicornis* restoration sites (left) and inshore, patch-reef massive coral restoration sites (right). Error bars represent one standard error uncertainties based on variability among transects within a given site. Note that the differences in the secondary (right), reef-accretion potential axis for offshore versus inshore patch reefs is due to the difference between the porosity of reef framework built by branching versus massive corals (see Methods).

Restoration of *Acropora cervicornis* 2–6 years following outplanting (Table 1) increased coral cover by 5% on average (± 0.68 standard error [SE]) across all offshore sites (LME_Site_: F_6,12_=1.92, *p* = 0.16), and resulted in significantly higher *A. cervicornis* cover at restored versus non-restored areas of the reefs (Fig. 2A; LME_Restoration_: F_1,53_=57.77, *p* < 0.001). The higher cover of *A. cervicornis* produced a significant, > 16-fold increase in gross carbonate production compared with non-restored areas (Fig. 2B and 3.62 ± 0.10 vs. 0.22 ± 0.47 kg CaCO_3_ m^− 2^ y^− 1^, on average; LME_Restoration_: F_1,53_=51.14, *p* < 0.001); however, we found that total number of outplants and time (years) since outplanting were both poor predictors of gross carbonate production (Fig. S1; Linear Regressions: *p* > 0.05). There was also no statistically detectable effect of site or the interaction between restoration and site (LME_Site_: F_6,12_= 1.94, *p* = 0.15; LME_Site*Restoration_: F_6,53_=1.58, *p* = 0.17).

In contrast to the significant impacts of *A. cervicornis* restoration offshore, there was no detectable impact of massive coral outplanting on coral cover at the inshore patch-reef sites (LME_Restoration_: F_1,27_<0.01, *p* = 0.95); average coral cover was ~ 9% at both restored and non-restored areas of those reefs (Fig. 2A). Likewise, gross carbonate production, which averaged 0.87 kg CaCO_3_ m^− 2^ y^− 1^ (± 0.05) at the patch reefs, was similar in restored and non-restored areas across the three sites (Fig. 2B; LME_Restoration_: F_1,27_= 1.36, *p* = 0.25; LME_Site_: F_2,2_= 1.55, *p* = 0.39); however, average total coral cover was significantly higher at the patch reefs compared with the offshore reefs (Fig. 2A; LME_Site_: F_9,14_= 7.60, *p* < 0.001; Tukey test: All patch reefs vs. Eastern Dry Rocks, *p* < 0.05; Cat’s Paw & Cook Island vs. Rock Key and Sand Key, *p* < 0.05; Cook Island vs. American Shoal, *p* < 0.05).

Total bioerosion averaged 0.86 kg CaCO_3_ m^− 2^ y^− 1^ (± 0.13) across all sites and there was no statistically detectable difference between restored and non-restored areas of the reefs (Fig. 2B; Paired t-test: t_9_ = 0.02, *p* = 0.98). With an average bioerosion rate of 0.61 kg CaCO_3_ m^− 2^ y^− 1^ (± 0.13), parrotfishes were the primary contributors to total bioerosion. Although bioerosion by parrotfishes at Sand Key was > 2x higher than the average elsewhere (Fig. 2B) due to several schools of large (fork length > 30 cm) *Sparisoma viride* observed during our surveys, that difference was not statistically detectable (LME_Site_: F_9,80_= 1.27, *p* = 0.27). Microbioeroders, urchins, and sponges contributed 0.22, 0.02, and 0.02 kg CaCO_3_ m^− 2^ y^− 1^ ( ± < 0.01 SE in all cases) to average bioerosion, respectively. Microbioerosion was significantly higher in non-restored areas (0.22 ± < 0.01 vs. 0.21 ± < 0.01 kg CaCO_3_ m^− 2^ y^− 1^, on average; LME_Restoration_: F_1,80_= 23.42, *p* < 0.001), likely because living calcifier cover is not included as erodible substrate in that calculation^35^ and was lower at Cat’s Paw compared with some offshore sites (LME_Site_: F_9,14_= 4.98, *p* = 0.004; Tukey Test Cat’s Paw vs. Eastern Dry Rocks, American Shoal, Rock Key, and Sand Key: *p* < 0.05). There were no statistically detectable differences in sponge or urchin bioerosion with restoration (LME_Restoration_: F_1,83_=0.01, *p* = 0.94 and LME_Restoration_: F_1,82_= 2.23, *p* = 0.14, respectively; and erosion by parrotfishes was only estimated at the site level), but urchin bioerosion was significantly higher at Cook Island compared to all other reefs except Cat’s Paw (both sites had relatively high abundances of *D. antillarum*^44^; LME_Site_: F_9,17_=6.83, *p* = 0.004; Tukey Test: *p* < 0.05) and estimated sponge bioerosion was significantly higher at Cat’s Paw compared with some offshore sites (LME_Site_: F_9,17_= 3.56, *p* = 0.01; Tukey Test: Cat’s Paw vs. Eastern Dry Rocks, American Shoal, and Sand Key: *p* < 0.05).

As a result of the higher carbonate production and similar rates of bioerosion, site level net carbonate production and reef-accretion potential was significantly higher at restored versus non-restored areas of the offshore reefs (Paired t-test: t_9_= -5.79, *p* = 0.001). In non-restored areas of most offshore reefs, reef-accretion potential was negative (Fig. 2C), averaging − 0.84 mm y^− 1^ (± 0.32) across sites; however, in areas where *A. cervicornis* was outplanted, average reef-accretion potential increased to 2.80 mm y^− 1^ (± 0.81), and was positive for all offshore reefs except Sand Key, where high estimated bioerosion by parrotfishes negated the positive impacts of restoration (Fig. 2). Reef-accretion potential was also positive—0.26 mm y^− 1^ (± 0.04), on average—at the patch-reef sites, but there was not a statistically detectable impact of restoration (Paired t-test: t_9_= − 0.85, *p* = 0.49).

### Structural complexity

**Fig. 3:**
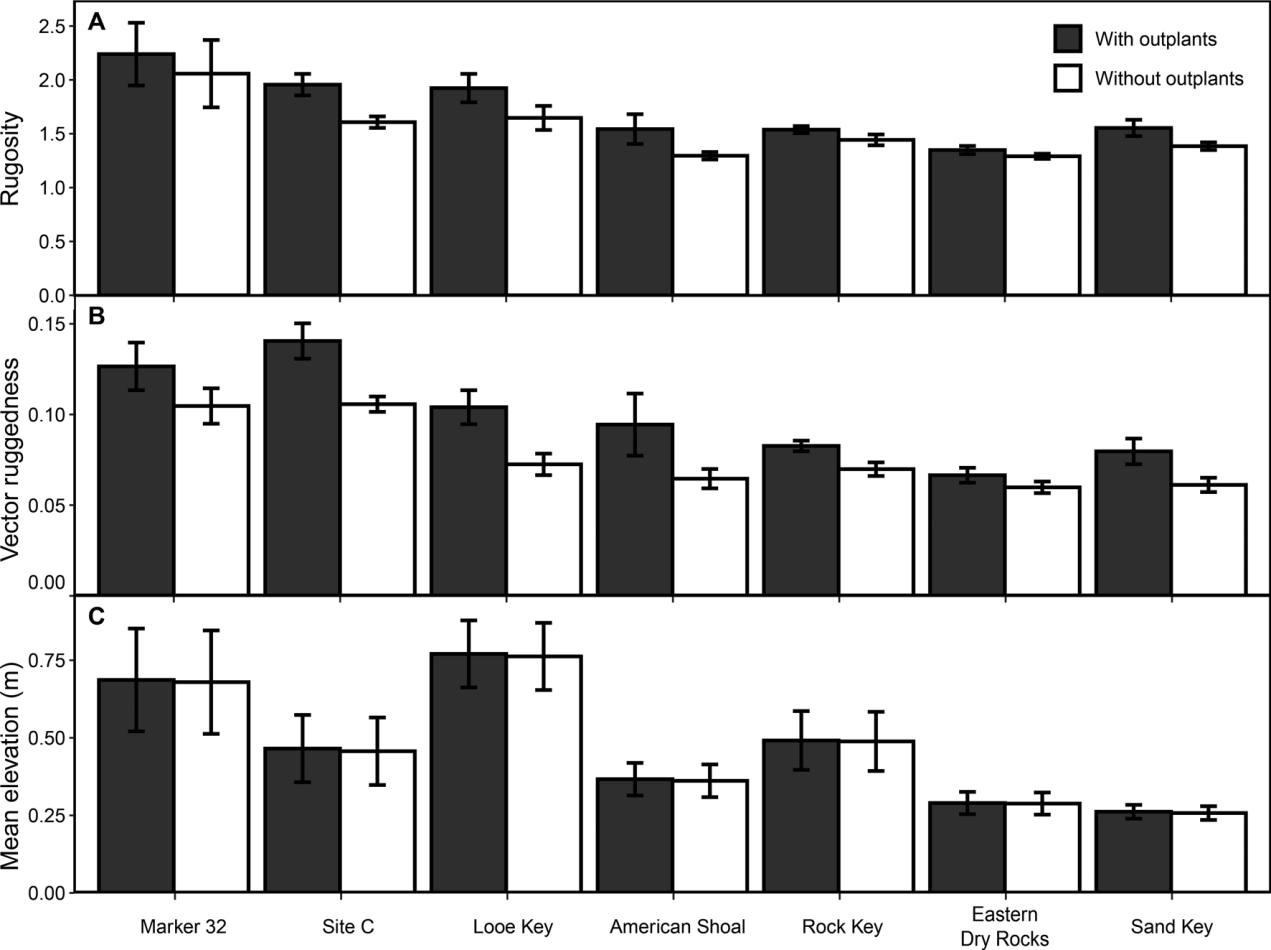
The impact of restoration on coral reef structural complexity. Comparison of three metrics of mean (bars) structural complexity—(**A**) rugosity, (**B**) vector ruggedness, and (**C**) mean reef elevation—at the offshore restoration sites based on structure-from-motion models with (filled bars) and without (open bars; outplants digitally removed from the models as described in the Methods) *A. cervicornis* outplants. Error bars represent one standard error uncertainties based on variability among transects within a given site.

The presence of *A. cervicornis* outplants resulted in a significant increase in rugosity and vector ruggedness (VRM) based on our Structure-from-Motion (SfM) models (see Methods; Fig. 3A and 3B; 1.72 ± 0.07 vs. 1.54 ± 0.06 [12%] and 0.10 ± < 0.01 vs. 0.08 ± < 0.01 [27%], respectively, on average; LME_Rugosity_: F_1,74_= 6.49, *p* = 0.01; LME_VRM_: F_1,74_= 23.06, *p* < 0.001); however the average increase in mean elevation of just 5 mm (1%) in restored areas was not statistically detectable (Fig. 3C; LME_Elevation_: F_1,74_=0.01, *p* = 0.92). There were significant, positive relationships between the percent cover of *A. cervicornis* and all three metrics of structural complexity (Fig. S2; LR_Rugosity_: F_1,38_= 111.90, *p* < 0.001, r^2^ = 0.74; LR_VRM_: F_1,38_=47.81, *p* < 0.001, r^2^ = 0.55; LR_Elevation_: F_1,38_=126.00, *p* < 0.001, r^2^ = 0.76), indicating that a 10% increase in *A. cervicornis* cover would result in a ~ 24% increase in rugosity (Rugosity = 2.37*A. cervicornis + 0.09), a ~ 47% increase in vector ruggedness (VRM = 3.97*A. cervicornis + 7.27), and a ~ 1 cm increase in mean reef elevation (Elevation [mm] = 0.95 *A. cervicornis + 0.27).

## Discussion

### Restoring the reef accretion process

Our study supports the conclusion that restoration of fast-growing, branching corals like *A. cervicornis* can have a rapid and significant impact on reef-accretion potential^25–27^. We found that in the absence of restoration, most of the offshore fore-reef habitats we surveyed in the Lower Florida Keys were net eroding (-0.84 mm y^− 1^, on average), supporting previous studies indicating that most of Florida’s reefs have net negative carbonate budgets^16,19^ and many are rapidly losing elevation^20,21,39^. In contrast, increases in *A. cervicornis* cover (5%, on average) 2–6 years following outplanting resulted in a > 16-fold increase in gross carbonate production, promoting neutral or positive reef-accretion potential (2.80 mm y^− 1^, on average) and significant increases in structural complexity at restored areas of the same reefs. Those impacts are modest compared with recent studies from the Indo-Pacific, where restoration of rapidly growing acroporids increased carbonate production rates to > 20 kg CaCO_3_ m^− 2^ y^− 1^ (refs^25,26^). ; however, our results are comparable to a recent study in southeast Florida, which estimated net carbonate production rates of ~ 3 kg CaCO_3_ m^− 2^ y^− 1^ (~ 3.2 mm y^− 1^, converted to reef-accretion potential as described in the Methods) 2–4 years following *A. cervicornis* outplanting, compared with net negative carbonate production where *A. cervicornis* was absent^27^.

These rapid impacts of restoration on the reef-accretion process are particularly remarkable in the context of the long-term marginality of reefs in south Florida. Regional reef development peaked ~ 8000–7000 years ago, when average rates of reef accretion were ~ 3 mm y^− 1^ (ref^6^). Florida’s subtropical climate has since become cooler and more variable^46,^ leading to declines in the rate and spatial extent of reef development, and culminating with a regional shutdown of reef accretion by ~ 3000 ago^6,12,47^. Carbonate-budget analysis of historical datasets from the Florida Keys demonstrated that the loss of reef-building corals from coral bleaching, cold-water stress, and disease over the last ~ 30 years drove further declines in reef-accretion potential from ~ 1 mm y^− 1^ in 1996 to < 0.1 mm y^− 1^ in 2022^16,17^, resulting in a regional transition to net erosion^19^. In just a few years, however, restoration of *A. cervicornis* effectively reversed this long-term declining trend on select areas of Florida’s reefs; on average, reef accretion-potential within restored areas was higher than the regional historical baseline in 1996, and, in many cases, higher than the geologic baseline from 8000–7000 years ago^6^ (Fig. 2C). Because erosion rates in our study did not differ significantly between outplanted and non-outplanted sites, the estimated increase in reef-accretion potential can be entirely attributed to the effect of enhanced *A. cervicornis* cover on carbonate production. Furthermore, because high-density “thickets” of *A. cervicornis* have been shown to deter grazing by parrotfishes^27,48^, our study, which did not separately quantify bioerosion by parrotfishes in restored and non-restored areas of the reefs, may underestimate the net effect of restoration on reef-accretion potential.

Our study also indicates that restoration of the reef-accretion process has the potential to reverse historical declines in reef structural complexity that have occurred throughout the western Atlantic since the 1970s^49^. These declines largely resulted from the loss of the unique habitats formed by *A. cervicornis* and its congener *A. palmata* following mortality from white-band disease in the late 1970s and early 1980s^50,^ highlighting why restoration of these ecosystem engineers is so essential. Whereas our estimates of rugosity in non-restored areas of the offshore reefs were similar to average western Atlantic rugosity measured since the 1980s of ~ 1.5^49^, restoration of *A. cervicornis* increased rugosity by 12% to 1.7. This positive trend is supported by a concurrent, 27%, increase in vector ruggedness (Fig. 3), and is similar to restoration-driven increases in rugosity observed in Indonesia and the Great Barrier Reef^25,26^. The rugosity of restored areas of Lower Keys reefs was not, however, as high as the > 2 rugosity index measured on some pristine western Atlantic reefs in the late 1960s and early 1970s^49^. Likewise, the minimal (~ 5 mm) increases in average reef elevation in restored areas were not statistically detectable and would do little to offset the decimeter-scale losses in elevation that have been observed in some parts of the Florida Keys since the 1930s^20^. Although the effects of *A. cervicornis* outplanting are not sufficient to fully restore historical reef structure, it is worth emphasizing that even modest increases in structural complexity can have substantial impacts on habitat availability for fishes and other reef associates and on reef hydrodynamics^22,23^, particularly on highly degraded reefs like those in south Florida^20,51^.

### The potential to restore ecosystem function

The significant, positive effects of *A. cervicornis* restoration on the reef-accretion process are promising, but there are several important reasons why the ability of coral restoration to meaningfully improve reef-scale ecosystem function into the future remains uncertain. First, although regional-scale restoration initiatives such as Mission: Iconic Reefs^41^ in south Florida are working to organize and expand restoration efforts, the spatial scales of most coral restoration activities to date have been relatively small (< 1 hectare)^29,33^. With an outplanting footprint of just ~ 200 m^2^ at any given location (subsite), the reefs surveyed in this study are no exception. As a result, those reefs likely remain net erosive on average, despite the significant increases in reef-accretion potential and structural complexity within the small outplanted areas. Producing reef-scale impacts on reef accretion and ecosystem function will require substantial scaling-up of coral restoration efforts^24,30^.

Second, the efficacy of coral restoration has proven to be highly spatially variable^29,31,33^. In inshore patch-reef environments, fast-growing acroporids are typically absent and reefs are instead built by slower-growing massive taxa such as *Orbicella* spp., *Siderastrea siderea*, and various brain-coral species^13^. Restoration practitioners have been able to jump-start the early growth of massive coral outplants through microfragmentation^52^; however, the near-term capacity of massive corals to produce new reef structure remains limited, as evidenced by the lack of a significant impact of massive coral restoration on reef-accretion potential in our study (Fig. 2C). Even at the offshore locations, the effects of *A. cervicornis* restoration were spatially variable (Fig. 2). The fact that we did not find a significant effect of either outplanting effort or time since outplanting on gross carbonate production (Fig. S1), suggests that site-specific differences in outplant mortality may be a primary driver of that variability^53^. Monitoring by Mote Marine Laboratory (see Methods) indicated that average survival of *A. cervicornis* at our sites 12 months after outplanting was above the 75% benchmark suggested by Schopmeyer et al.^54^ (85% ± 3.0%), and there were at least 10-fold increases in total linear extent of colonies after 3–5 years; however, survival rates were variable among sites (51–98% after 12 months; Table S1). Although we did not find a significant relationship between those survival statistics and gross carbonate production at the subsites (Fig. S3), we observed significant evidence of recent partial mortality from snail predation and disease (especially in 2023^44,55^) that may not have been captured by the sort of periodic monitoring conducted by most coral restoration programs^32,33^.

The third, and most challenging question is whether the positive impacts of restoration can persist long term. Even if ongoing restoration can maintain living populations of *A. cervicornis* on Florida’s reefs in the coming decades, the ability of restored reefs to achieve the maximum level of key functions, such as wave-breaking during storms, will also depend on whether reef accretion can keep pace with future sea-level rise^43^. The ~ 3 mm y^− 1^ average rates of reef-accretion potential estimated for restored areas of Florida’s reefs^27^ (Fig. 2C) are comparable to the present-day rate of sea-level rise in Key West of 2.6 mm y^− 1^ (https://tidesandcurrents.noaa.gov/sltrends/; Station ID: 8724580), but would be too slow to keep pace with even moderate projections of regional sea-level rise predicted for the coming decades (i.e., ~ 0.63 m by 2100 with 0.5 m of global sea-level rise, analogous to shared-socioeconomic pathway [SSP] 4.5^56,57^). Estimated carbonate production rates were, however, found to be significantly higher, > 10 kg CaCO_3_ m^− 2^ y^− 1^ (~ 11 mm y^− 1^), in naturally occurring *A. cervicornis* “thickets” in southeast Florida, where average *A. cervicornis* cover approached 30%^27^. If restoration could be scaled up to that level, reefs could potentially keep pace with projected local sea-level rise under SSP4.5, but not with the > 1 m of sea-level rise projected by the end of the century under a worst-case emissions scenario (SSP8.5)^43,56,57^.

It is also worth emphasizing that reef-accretion *potential* estimated with carbonate-budget modeling is likely to be a high-end estimate of realized future reef growth^8^. Carbonate budgets only incorporate estimates of reef-framework erosion by bioeroders and do not consider the impacts of chemical dissolution or physical erosion^58^, both of which may have a significant impact on the reef-accretion process in south Florida^36–39^. Moreover, the relatively fragile morphology of *A. cervicornis* makes its skeletons especially vulnerable to fragmentation and transport, particularly during storm events^59,60^. The high potential for export of *A. cervicornis* skeletons out of the reef framework suggests that its realized contribution to long-term reef accretion may be substantially lower than our models of reef-accretion *potential* predict. Indeed, despite the high abundance of *A. cervicornis* observed on reefs in the Florida Keys historically, *A. cervicornis* skeletons made up < 3% of the regional reef framework built during the last ~ 8000 years^6^. Overall, our results suggest that although restoration of *A. cervicornis* can rapidly produce new reef habitat that could have valuable impacts on associated reef fauna in the short term^22,23^, the potential of *A. cervicornis* to impact large-scale and long-term reef function remains uncertain.

### Impacts of the 2023 bleaching event

The potential for coral restoration to rise to the challenge of maintaining future reef function is particularly uncertain in the context of projected increases in the frequency and severity of coral-bleaching events under climate change^40,42,61^. Climate change is predicted to decrease carbonate production by at least 150% globally by 2100 (under SSP 4.5), primarily due to the increases in coral-bleaching-related mortality^40^. Many inshore patch reefs in the Florida Keys already experience moderate annual bleaching^62,63^ and annual severe bleaching is expected to affect reefs regionwide before 2050^61^. Without significant thermal acclimatization of corals, carbonate production in south Florida is likely to experience significant declines in the near future, even with aggressive upscaling of coral-restoration efforts^42^. The acroporid corals that have been the most commonly outplanted taxa in the western Atlantic to date due to their rapid growth rates and high functional value for creating complex reef habitats^24,43,54,60,64^ are also particularly sensitive to thermal stress^65,66^. The vulnerability of acroporids to bleaching-related mortality was brought into stark focus in the summer and fall of 2023, when the Florida Keys experienced the most severe thermal stress event on record^63,67^.

The onset of the 2023–2024 El Niño event brought record high ocean temperatures globally and widespread marine heatwaves during the summer of 2023^68^. In the Lower Florida Keys where our study took place, accumulated thermal stress above critical bleaching thresholds (i.e., 4 degree-heating weeks [DHW] for bleaching and 8 DHWs for bleaching-related mortality) was detected by late July: more than a month earlier than the typical peak of regional thermal stress. Unprecedented thermal stress persisted for more than three months, with offshore reefs in the Lower Keys experiencing > 17 DHWs and inshore patch reefs experiencing > 20 DHWs at the peak of the event^67^. By the end of the summer, 100% of reefs in the region were experiencing some level of coral bleaching^62,63^.

Coral bleaching was just beginning in mid-July of 2023 as the reef surveys for this study were being completed. Although bleaching and paling were observed at the inshore patch reefs, all corals were still alive at the time of our surveys, and significant coral bleaching had not yet begun at the offshore sites we visited^44,55^. By the end of the event, however, outplanted corals throughout the Florida reef tract had experienced significant levels of mortality, with the most severe impacts on acroporids in the Lower Keys^63,69,70^. Post-bleaching assessments (see Methods) indicated that overall survival of *A. cervicornis* fragments outplanted by Mote Marine Laboratory averaged 1.0% (± 0.4) across all sites surveyed (the majority of which were in the Lower Keys; three in the Middle Keys), and 0.1% (± 0.1) at the sites surveyed in this study (Table S1). Survival of *A. palmata* across Mote’s outplanting sites was even lower at 0.6% (± 0.1), on average (*A. palmata* was not outplanted at the sites surveyed in this study). Likewise, nearly 80% of wild *A. palmata* genotypes were lost in the upper Florida Keys and *A. palmata* became locally extinct in the Dry Tortugas National Park following the 2023 bleaching event^69,70^. Massive coral outplants fared much better, however, with survival of 59.2% (± 2.9) across Mote’s patch-reef sites and 64.1% (± 11.7) at the Cat’s Paw and Dog’s Leg sites surveyed in this study (data were not available from Cook Island; Table S1). Survival rates were highest for outplanted *Montastraea cavernosa* and *O. faveolata*—74.7% (± 5.4) and 70.9% (± 4.3), respectively—and lower for outplanted brain corals (e.g., 52.5 ± 7.3% for *Pseudodiploria clivosa*, the brain coral species with the most post-bleaching data). Although Neely et al.^63^ found that mortality of wild massive coral colonies was < 0.5% throughout the Florida Keys, both overall and species-specific mortality rates of wild massive corals within our study area in the Lower Keys were similar to outplant mortality rates observed by Mote.

**Fig. 4:**
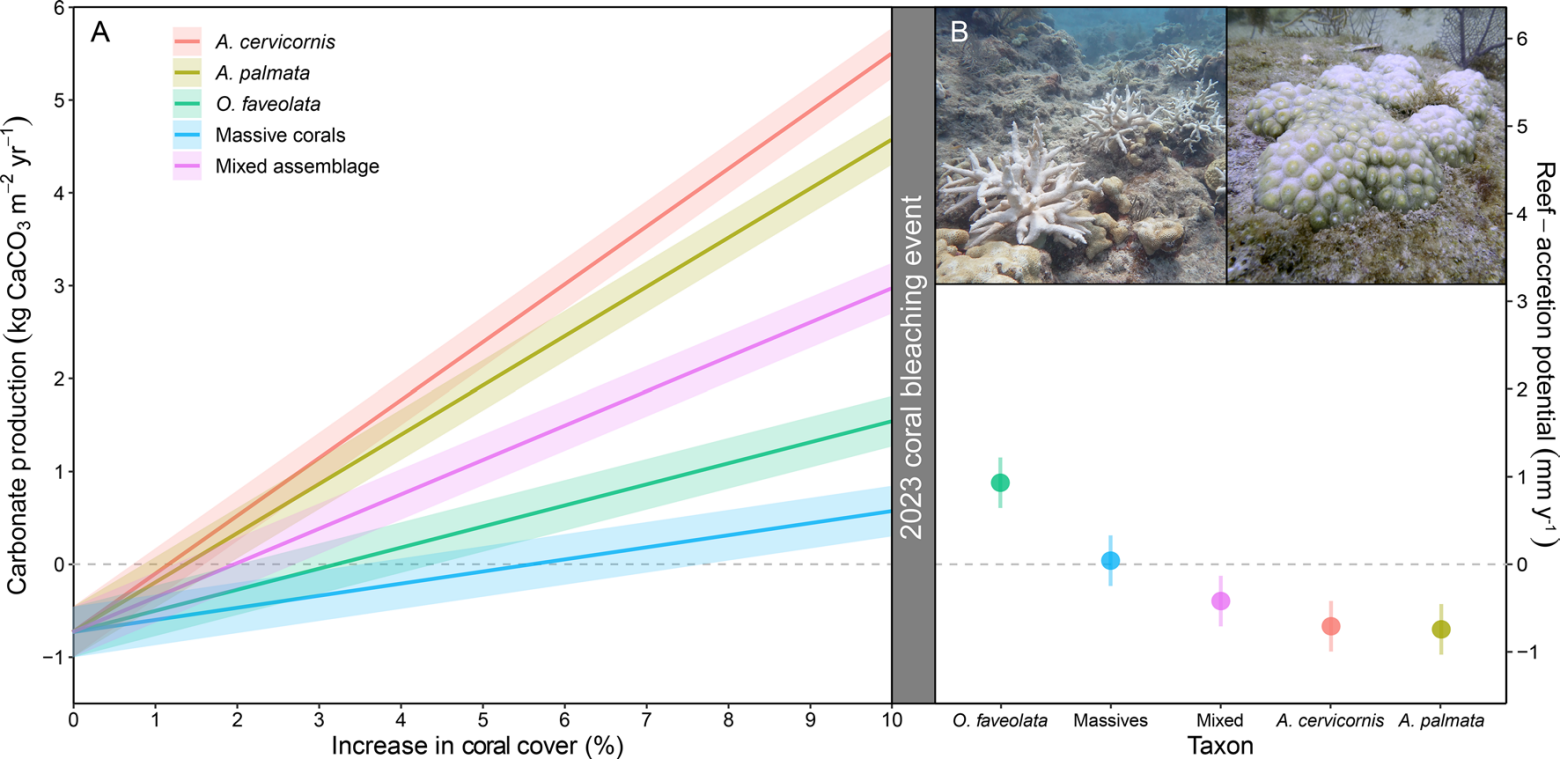
Illustration of the trade-offs between different strategies for restoring the reef-accretion process in the context of severe coral-bleaching impacts. (**A**) Projected mean (solid lines; ± standard error [SE; shading]) increases in net carbonate production and reef-accretion potential of offshore reef sites in the Lower Florida Keys under different restoration scenarios. The baseline of the projections (0% increase in percent cover) corresponds to the means (± SEs) of non-restored areas of the offshore reefs surveyed in our study. Assuming that bioerosion remained constant, we used species-specific mean (± SE) calcification rates^71^ to estimate increases in net carbonate production and reef-accretion potential with increases in percent cover of corals. The “massive corals” scenario is based on the average of the calcification rates for all massive taxa outplanted by Mote: *Orbicella* spp., *Montastraea cavernosa*, *Pseudodiploria* spp., and *Diploria labyrinthiformis* (see Methods). The “mixed assemblage” is based on the relative percent cover of massive corals, *Acropora cervicornis*, and *A. palmata* under a new outplanting strategy being employed by Mote at some sites: 41% massive corals, 4% *A. cervicornis*, 55% *A. palmata*. (**B**) Conceptual model of the impact of the severe 2023 bleaching event based on species-specific mean (± SE) survival rates of outplanted coral measured by Mote throughout the Florida Keys (Methods; Table S1). Photographs show the severe bleaching of *A. cervicornis* and more moderate bleaching of massive, *M. cavernosa*, corals. Photos taken during the 2023 bleaching event by Z. Craig (left) and E.A. Lyons (right).

The differential impacts of the 2023 regional bleaching event on massive versus branching corals suggests that there are important trade-offs between coral growth rates and the probability of coral survival that are worth considering as coral-reef managers confront the challenge of restoring coral reefs under climate change. We created a conceptual model of those trade-offs using carbonate-budget modeling. We first quantified the theoretical impacts of different restoration scenarios on reef-accretion potential at the offshore reefs in our study by assuming restoration-driven increases in percent cover of different combinations of outplanted coral taxa from 0 to 10% (Fig. 4A; Methods). We then estimated how differential bleaching-related mortality would affect the ability of those reefs to maintain positive reef-accretion potential by reducing coral cover in the 10% scenario by the observed mortality rates of *A. cervicornis*, *A. palmata*, *O. faveolata*, and all massive coral taxa during the 2023 bleaching event (Fig. 4B; Methods). As expected, in the absence of bleaching-related mortality, increasing the cover of branching acroporids would have the most substantial and rapid impact on reef-accretion potential^16,25–27,43^ (Fig. 4A); however, near-total mortality of restored acroporids following the 2023 bleaching event has likely largely eliminated their positive impacts (Fig. 4B). If previously unprecedented levels of thermal stress, like those that affected the Florida Keys in 2023, become more common, then restoration of slower growing, but more resilient massive corals, may prove more effective long term^63^ (Fig. 4B). The relatively high (> 70%) survival of *O. faveolata* following the 2023 bleaching event^63^ suggests it may be a particularly promising target for restoration moving forward. Although this taxon calcifies > 2x slower than acroporids, it calcifies ~ 2x faster than other massive taxa commonly outplanted in the western Atlantic^71^. Moreover, its skeletons are more robust and more likely to persist long term in the reef framework than those of *A. cervicornis*, which is why *Orbicella* spp. have been dominant contributors to regional reef-building for millennia^6,15,16^. Bleaching is, of course, not the only source of coral mortality, and species-specific differences in the susceptibility of corals to disease (e.g., stony coral tissue loss disease, white-band disease), hurricane impacts, and corallivory^17,50,63,72,73^ could also significantly impact long term restoration success. With the high likelihood of increases in the sources and severity of disturbances moving forward, it may be beneficial for restoration practitioners to adopt a “bet hedging” strategy, by simultaneously outplanting a “mixed” (Fig. 4) assemblage of corals including both fast-growing acroporids that can rapidly increase reef-accretion potential, as well as more resilient massive corals that may be more likely to persist long term^27^. This approach, wherein outplanting more closely reflects natural baselines of coral species diversity, could help to future-proof restoration efforts and optimize functional outcomes until the threat of climate change is mitigated.

### Quantifying future restoration success

Over the last several decades, the field of coral-reef restoration has grown exponentially, from a series of haphazard efforts to address small-scale disturbances^74^ to an integrated, global-scale community working to stem the tide of unprecedented coral-reef degradation^24,30^. As the scale of coral-reef restoration has grown, so too has the hope that restoration can be effective not only in maintaining populations and diversity of imperiled corals, but also in meaningfully affecting important reef processes and functions^30^. Despite the breadth of restoration goals, limited resources have made consistent and effective post-restoration monitoring challenging^32,33^. Indeed, the fact that we were unable to detect a significant relationship between the commonly quantified metrics of outplanting effort, outplant survival, or colony growth and carbonate production (Figs. S1, S3), indicates that these traditional monitoring metrics may fail to capture the broader impacts of restoration on critical geo-ecological processes^3^. If the ultimate goal of coral-reef restoration is to revive ecosystem processes and function^30^ then the efficacy of restoration activities in enhancing reef accretion and habitat complexity provides the most fundamental measure of restoration success.

Our study demonstrates that coral-reef carbonate budget modeling and measurements of habitat complexity using SfM can provide efficient and effective mmeasure of restoration success that go beyond traditional monitoring metrics to evaluate the effect of restoration on ecosystem process and function (Figs. 2 and 3). The basic methodology for developing carbonate-budget models is well-established^34,35^ and estimates of coral-reef carbonate production can now be automatically generated from traditional point-count surveys of reef imagery (e.g., by using the built-in functions in CoralNet^71,75^ https://coralnet.ucsd.edu/). Collecting that monitoring imagery using cost-effective SfM methodologies^76^ would enable researchers to use those same data to generate estimates of corresponding changes in reef complexity (Fig. 3). In regions like South Florida, where extensive SfM imagery datasets already exist, researchers can leverage this technology to track changes over time, model future scenarios, and implement targeted restoration strategies^77^. Regional-scale rates of bioerosion are likewise well-constrained in south Florida^16,19^ allowing for the possibility of inputting generalized estimates of bioerosion into carbonate-budget models to evaluate trade-offs between various restoration scenarios being considered by practitioners^16^ (Fig. 4). These models could be further improved by integrating them with regional-scale data on coral growth and reef erosion^20,21,36,39,60,64,78^ and by incorporating existing restoration monitoring data to project population growth rates under various disturbance scenarios^42,43^.

Coral-restoration practitioners are faced with the daunting challenge of determining how to utilize limited resources to ensure the persistence of coral-reef ecosystems under the threat of accelerating and compounding local- and global-scale disturbances. There are, of course, important uncertainties associated with the carbonate-budget and SfM models we have described, and no model can fully constrain the complex geo-ecological functions of coral-reef ecosystems^16,58^; however, given the urgency of the ongoing coral-reef crisis, models like these that can help managers make informed decisions about how to optimize the functional outcomes of coral-reef restoration could prove essential to maintaining critical coral-reef habitats and the invaluable ecosystem services they provide.

## Methods

### Description of restoration sites

At the time of our study, Mote had outplanted ~ 240,000 coral fragments throughout the Florida Keys and ~ 15,000 at our study sites alone. The primary coral species outplanted at the eight offshore reefs we surveyed was *A. cervicornis*. Restoration at the three inshore patch-reef locations instead focused on a variety of massive coral taxa that were historically common on those reefs (i.e., *Orbicella* spp., *Pseudodiploria* spp., *M. cavernosa*, and *Porites astreoides*). The corals were generally outplanted in multi-fragment, monogenetic clusters (asexual clones) with the goal of rapidly producing a sexually mature colony size when the fragments fuse (see https://www.the-scientist.com/restored-corals-spawn-hope-for-reefs-worldwide-68368). The average footprint of the outplanted areas (subsites) of the reefs was ~ 200 m^2^ and average outplant density was ~ 2.5–5 coral clusters m^− 2^, with five *A. cervicornis* or 15–20 massive coral fragments typically outplanted in each cluster.

During the summers of 2022 and 2023, we conducted surveys at outplanted or “restored” (*n* = 4–9) and non-outplanted or “non-restored” (*n* = 4–6) areas within subsites of each of the eight offshore reefs and three inshore patch reefs (sites; Table 1) to quantify the following functional metrics: (1) gross carbonate production, (2) bioerosion by parrotfishes, sponges, urchins, and microbioeroders, (3) net carbonate production and reef-accretion potential, and (4) structural complexity. For restored transects, divers identified areas of the reef with a high density of outplanted corals and haphazardly placed transects within those areas. Each transect was separated by ~ 2 m. Because we selected for areas of high outplant density, the data from restored transects are likely not representative of reef-scale impacts of restoration, but rather a measure of the direct, small-scale impacts of outplanting. Non-restored transects, which were used to quantify the baseline carbonate budgets of the reefs without the impact of restoration, were haphazardly placed on nearby areas of the reef (i.e., adjacent spurs or ledges for offshore reef sites) where no outplants were present.

### Structure-from-motion surveys

At each site, except for Summerland Ledges, photographic surveys were conducted along 10–12, 10 × 2 m belt transects by SCUBA divers using a downward facing Canon Powershot S120 camera in an underwater housing set to collect images in RAW format in continuous shoot mode (Table S2). The number of transects varied per subsite depending on the availability of restored or control reef areas. Imagery at all other sites was collected 1–2 m above the reef using a double-lawnmower swim pattern ensuring 70–80% forward and lateral overlap between images (Table S2). Prior to image acquisition, divers used a metric survey tape to delineate the sampling area then placed 3–4 coded 25-cm scalebar targets evenly throughout the transect areas to provide accurate scale for developing SfM models. Summerland Ledges is an experimental restoration site consisting of two 10 x 10 m restored areas and one 10 x 10 m control plot. The two restored plots at Summerland Ledges were surveyed 2 m above the reef using a dual Nikon D7000 DSLR camera system and the control plot was surveyed using a Canon EOS R from the water’s surface (~5 m above the reef). All imagery collected for this study are available in a U.S. Geological Survey data release^55^.

SfM data products (point clouds, digital surface models [DSMs], and orthomosaics) were generated using Agisoft Metashape Pro v2.0 or later generally following established protocols^79^. A detailed description of the SfM workflow and settings are provided in the Electronic Supplementary Material (Table S2; Fig. S4). Briefly, the images from each transect were checked for proper orientation then uploaded into Metashape. Three-dimensional models were then generated using the following basic steps: (1) align images into a sparse point cloud, (2) scale the model and run initial bundle adjustment for lens calibration, (3) reduce the number of low quality points in the model to improve accuracy, (4) orient and position the model in a local coordinate system, (5) generate dense three-dimensional point cloud, (6) segment the point cloud into four classes: noise, reef base, canopy (i.e., gorgonians), and outplants, (7) filter the point cloud based on segmentation classes to generate DSMs, (8) render orthomosaics and (9) export all SfM data products. All SfM data products are also available in a U.S. Geological Survey data release^44^.

### Carbonate budgets

We used reef-census data to estimate gross carbonate production, bioerosion, net carbonate production, and reef-accretion potential following an adaptation of the ReefBudget v2 methodology^16,35^ (https://www.exeter.ac.uk/research/projects/geography/reefbudget/*).* Percent cover of calcifying reef taxa (i.e., corals and crustose coralline algae) and other benthos was quantified by conducting point-count analysis of a 10 × 1 m belt transect extracted from the two-dimensional SfM orthomosaics described above using the online software CoralNet (https://coralnet.ucsd.edu/*).* Ten non-overlapping 1 × 1 m images were extracted from each orthomosaic and the benthos beneath each of 150 points were identified in each image. All coral taxa were identified to species, except for *Orbicella*, *Pseudodiploria*, and *Millepora* spp. which were pooled by genera. Other benthos, including crustose coralline algae, macroalgae, sponges, gorgonians, zoanthids, consolidated bare substrate (which was generally covered in turf algae), and unconsolidated substrate (sand and rubble) were identified categorically.

The percent cover of each reef calcifier was multiplied by area-normalized taxon-specific calcification rates and summed for each transect to quantify gross carbonate production (in kg m^− 2^ y^− 1^) using the built-in function in CoralNet^71,75^. We estimated microbioerosion by multiplying the total area of consolidated reef substrate not occupied by calcifying taxa (from the point-count analysis) by the western Atlantic mean microbioerosion rate of 0.24 kg m^− 2^ y^− 1^ (ref^35^).

We also conducted surveys of bioeroding parrotfishes, sponges, and urchins following the ReefBudget v2 protocol^35^. Divers recorded the number and size of bioeroding sponges (*Cliona aprica*,* C. caribbaea*,* C. tenuis*,* C. varians*,* C. delitrix*, and *Siphonodictyon coralliphagum*) and bioeroding urchins (*Diadema antillarum*,* Echinometra lucunter*,* Ec. viridis*, and *Eucidaris tribuloides*) along the same 10-m transects surveyed for SfM (sponges: 10 × 1 m belt transects; urchins: 10 × 2 m belt transects). Because parrotfishes are highly mobile, we assumed that their densities did not vary significantly between restored and non-restored areas of the reefs. Therefore, we recorded the species, size (fork length), and life phase (initial or terminal phase) of bioeroding parrotfishes (*Sparisoma viride*, *Sp. aurofrenatum*, *Sp. rubripinne*, *Sp. chrysopterum*, *Scarus vetula*, *Sc. taeniopterus*, *Sc. iseri*, *Sc. guacamaia*, *Sc. coeruleus*, and *Sc. coelestinus*) within 8–10 larger (25 × 4 m) belt transects to estimate site-level bioerosion by parrotfishes. The abundances of each functional group of bioeroders were multiplied by the species-, size-, and, for parrotfishes, life-phase-specific bioerosion rates suggested in ReefBudget v2^35^ and summed to estimate total bioerosion.

Total bioerosion at each restored and non-restored area of each site was subtracted from gross carbonate production to estimate net carbonate production. We converted net carbonate production to estimates of reef-accretion potential based on the following Equation^80^:

$$\:Reef\:accretion\:potential=\frac{Net\:carbonate\:production}{p(1-porosity)}$$

where *p* is the density of calcium carbonate^82^ (2.9 g cm^− 3^) and *porosity* was estimated from cores of geologic reef framework in the Florida Keys built by branching (*A. palmata*) or massive corals^81^: 67.5% (± 3.7) and 55.8% (± 1.9), for offshore and patch-reef sites, respectively. Porosity of *A. palmata* framework was used in place of *A. cervicornis* as the latter taxon was rare in the reef framework of the Florida Keys^15^. We emphasize that reef-accretion potential provides a high-end estimate of the likely realized reef-accretion rate because it does not incorporate non-biological sources of physical erosion or chemical dissolution^58^. It nonetheless provides a robust metric for quantifying the relative state of the reef-accretion function and can offer valuable information for coral-reef management^16,43^. All carbonate budget data are available in a U.S. Geological Survey data release^44^.

### Structural complexity

Although we did not expect restoration of relatively flat, massive coral outplants to significantly change reef structural complexity at the inshore patch-reef sites, we hypothesized that *A. cervicornis* would increase structural complexity at the offshore sites. To test that hypothesis, we evaluated three structural complexity metrics—mean elevation, rugosity, and roughness—based on 1-cm resolution exports of the DSMs from the SfM models of the offshore reefs. Mean elevation of each DSM was calculated relative to the lowest point of each transect model. Rugosity, which is typically measured in situ as the ratio of the length of a chain draped over the contours of reef substrate to the linear distance of reef measured, is the most widely used metric of structural complexity^82–84^. We measured the overall rugosity of each DSM by quantifying the ratio of the three-dimensional surface area to the two-dimensional planar surface area of the transect^83–85^.

Because typical rugosity measurements can be influenced by reef slope^86,87^ we also used the Multiscale DTM package in R^88^ to calculate complexity measures that separate slope variability from surface irregularities and allow calculations at multiple spatial scales. Before conducting terrain analysis, we selected transects with diverse profile shapes (flat, sloped, concave, convex, ledge, hills, and valleys). We then compared roughness metrics from 1-cm resolution DSM exports using various window sizes (3 × 3, 5 × 5, 7 × 7, and 11 × 11 cm). A 5 × 5 cm neighborhood window size was determined to be the most appropriate size to reduce noise without loss of topographic details and patterns. The DTM package calculates 15 metrics encompassing terrain attributes from five common terrain groups: slope, aspect, curvature, relative position, and roughness. For this study, we focused on roughness, defined as topographic variability or surface “bumpiness”. The package includes four roughness metrics: arc-chord corrected surface area to planar area ratio, vector ruggedness, adjusted standard deviation, and the roughness index-elevation^88^; however, because we found that these metrics were highly correlated for our DSMs (Fig. S5), we used vector ruggedness as a representative metric of roughness in our analysis. This metric measures surface orientation variability by comparing normal vectors within an area to those in neighboring areas, with values from zero (smooth) to one (rough)^89^. The structural complexity data are available in a U.S. Geological Survey data release^44^.

### Statistical analysis

All statistical analyses were conducted in RStudio^90^ (v.4.3.1). We statistically compared coral cover, gross carbonate production, and bioerosion by sponges, urchins, and microbes at restored versus non-restored areas of the reefs with linear mixed-effects models (LMEs) using the *nlme* package^91^. Restoration status (restored vs. non-restored) and site were treated as fixed factors in the models and subsite as a random intercept. The results of the models were summarized using the *anova* function and pairwise differences among sites were evaluated using the *emmeans* package^92^. Because of the differences in the species outplanted and the timing that restoration was initiated, gross production was analyzed separately for offshore and inshore reefs and *A. cervicornis* cover was analyzed for the offshore sites only. Additionally, because the restoration design and effort were different at Summerland Ledges than atcompared with the other offshore sites, and average *A. cervicornis* cover there was < 1%, that site was excluded from our statistical analyses. The site-level estimates of total bioerosion and reef-accretion potential for restored and non-restored areas of the reefs were compared using paired t-tests (*t.test* function). Whereas the comparison of total bioerosion included all sites, separate t-tests were used to compare reef-accretion potential at offshore and inshore patch reefs. Levene’s tests were conducted to ensure the assumption of homogeneity of variances was met (*p* > 0.05). Finally, we used linear regression analysis (*lm* function) to evaluate whether total number of outplants, time since outplanting began, percent survival 12 months after outplanting, or total linear extent 3–5 years after outplanting were significant predictors of *A. cervicornis* cover or gross carbonate production at the offshore survey locations (i.e., each subsite). Examination of residuals plots suggested that the residuals of the models approximately conformed to the assumption of normality.

To account for the possibility that there were differences in structural complexity of the restored and non-restored areas of the offshore reefs that were not related to restoration (i.e., pre-existing differences in the geological structure), we did not compare restored and adjacent, non-restored transects for the structural complexity analysis. Instead, we more directly evaluated the impact of restoration on structural complexity by using LMEs to compare the three structural complexity metrics (rugosity, vector ruggedness, and mean elevation) calculated using the complete three-dimensional models of the transects (only noise removed; “no filter”) to models where the outplanted *A. cervicornis* colonies were digitally removed from the models of restored transects (outplant filter). Filter was treated as a fixed effect and site was treated as a random intercept in the models. The results of the models were summarized with the *anova* function and pairwise differences among sites were evaluated using the *emmeans* package^92^. Finally, we evaluated the relationship between outplanted *A. cervicornis* cover and the percent increase in rugosity and vector ruggedness and the absolute increase in elevation using linear regression analysis (*lm* function). Examination of residuals plots suggested that the assumption of normally distributed residuals was met for all models after removing a significant outlier from Marker 32 (restored transect six) that had high, 22%, *A. cervicornis* cover but only moderate changes in structural complexity.

### Evaluating restoration trade-offs

We used the carbonate budgets from our study to evaluate how reef-accretion potential would change under different restoration scenarios with and without bleaching-related mortality. We used the average (± SE) reef-accretion potential from non-restored areas of the eight offshore reefs in our study, -0.84 mm y^− 1^ (± 0.32), as the baseline of the models (i.e., + 0% coral cover). We then calculated idealized estimates of how reef-accretion potential would change with 1% increases in coral cover up to + 10% achieved by outplanting of (1) *A. cervicornis*, (2) *A. palmata*, (3) *O. faveolata*, (4) various massive corals, or (5) a mixed assemblage that includes both acroporids and massive taxa. Those estimates were calculated by adding the product of the proportional increase in coral cover and species-specific mean (± SE) calcification rates^71^ of outplanted taxa to baseline net carbonate production of the sites and converting the resulting value to reef-accretion potential as described previously. The “massive corals” scenario used the average of the mean (± SE) calcification rates for all massive taxa outplanted by Mote: *O. faveolata*, *M. cavernosa*, *Pseudodiploria* spp., and *D. labyrinthiformis*^71^: 13.03 kg CaCO3 m^− 2^ y^− 1^ (± 2.08). The “mixed assemblage” scenario is based on the relative percent cover of massive corals, *A. cervicornis*, and *A. palmata* under a new outplanting strategy being employed by Mote at some sites: 41% massive corals, 4% *A. cervicornis*, 55% *A. palmata*. This scenario also used the combined massive coral calcification rate in addition to the species-specific calcification rates for *A. cervicornis* and *A. palmata*.

We then evaluated how a severe coral-bleaching event, like the one that affected the Florida Keys in 2023, would reduce restoration-driven increases in reef-accretion potential under the five outplanting scenarios. For that model, we decreased the maximum outplant coral cover (+ 10%) based on taxon-specific survival rates of corals following the 2023 bleaching event (i.e., adjusted increase in coral cover = 10% x survival rate). The adjusted maximum outplant coral cover was then multiplied by the scenario-specific calcification rate and added to the baseline model, as described above. To determine outplant survival after the 2023 bleaching event, Mote researchers conducted post-bleaching surveys at 359 subsites (41 reefs) in the Lower (38 reefs) and Middle Keys (3 reefs) where *Acropora* spp. were outplanted and 40 subsites (12 reefs) in the Lower Keys where massive corals were outplanted. Survival rates of taxon at each subsite was calculated by dividing the number of outplanted coral fragments with living tissue by the total number of fragments initially outplanted, which is also how 12-month survival rates were calculated (Table S1). The average survival rates for *A. cervicornis*, *A. palmata*, *O. faveolata*, and massive corals overall following the 2023 bleaching event were 1.03% (± 0.41), 0.58% (± 0.13), 70.94% (± 4.28), and 59.17 (± 2.92), respectively.

## Electronic supplementary material

Below is the link to the electronic supplementary material.

Supplementary Material 1.

## Data Availability

All data including reef imagery (10.5066/P1WHKTRD), Structure-from-Motion data products, reef census data, carbonate budgets, and reef structural complexity data are available in U.S. Geological Survey data releases (10.5066/P13HMEON).
