# Supplementary material for: Coral restoration can drive rapid increases in reef accretion potential
Source: Sci Rep. 2025 Aug 4;15:28353. doi: 10.1038/s41598-025-04818-3 (PMC12322247; doi:10.1038/s41598-025-04818-3)
Supplement: Supplementary file 1 — Supplementary Material 1. [file 41598_2025_4818_MOESM1_ESM.pdf]

Electronic Supplementary Material

for

Coral restoration can drive rapid increases in reef accretion potential

Lauren T. Toth[1*], Selena A. Johnson[1], Erin O. Lyons[2], Jason Spadaro[3], Anastasios Stathakopoulos[1], Sierra Bloomer[1], Jennifer Mallon[4], Connor M. Jenkins[1], Sara D. Williams[5], Ian Combs[3], Zachary Craig[3,6], Erinn Muller[5]

[1]U.S. Geological Survey, St. Petersburg Coastal and Marine Science Center, St. Petersburg, FL

[2]Cherokee Nations System Solutions, Tulsa, OK

[3]Mote Marine Laboratory, Summerland Key, FL

[4]National Coral Reef Institute, Halmos College of Arts and Sciences, Nova Southeastern University, Dania Beach FL

[5]Mote Marine Laboratory, Sarasota, FL

[6]Division of Aquatic Resources, Hawai'i Department of Land and National Resources, Kailua-Kona, HI

*Corresponding author: ltoth@usgs.gov

Disclaimer: Any use of trade, firm, or product names is for descriptive purposes only and does not imply endorsement by the U.S. Government.

Table S1. Table with data on the survival and growth of outplanted corals collected by Mote Marine Laboratory researchers at surveyed subsites. TLE=total linear extent. "-" indicates that no data were available. Patch-reef sites with N/A for 3–5 Year TLE had not been outplanted for five years at the time of this study, so those values are not applicable to those sites. *The M32_AP3 site was outplanted by the Coral Restoration Foundation, not Mote Marine Laboratory.

Site	Subsite	# Outplants	Year Outplanted	1-Month Survival	12-Month Survival	3–5 Year TLE	Post-2023 Bleaching Survival
Sand Key	SK_S2	1000	2016	70.8%	68.4%	230.7%[5]	0.00%
	SK_S5	500	2018	96.4%	93.4%	1305.8%[3]	0.00%
	SK_S7	500	2019	97.6%	93.0%	1521.4%[3]	0.00%
	SK_S12	500	2021	99.8%	78.6%	-	0.00%
Rock Key	RK_R1	1000	2016	95.6%	87.4%	2013.5%[5]	0.00%
	RK_R2	1000	2016	99.5%	97.8%	1465.5%[5]	0.00%
Eastern Dry Rocks	EDR_T1	1000	2016	95.20%	89.1%	699.1%[5]	0.00%
	EDR_T3	500	2019	96.0%	90.4%	1063.4%[3]	0.00%
	EDR_T8	500	2020	87.4%	71.0%	125.9%[3]	0.00%
Marker 32	M32_1	600	2019	100.0%	93.2%	1298.9%[3]	0.00%
	M32_AP3*	500	2021	-	-	-	-
American Shoal	AS_Q4	550	2018	90.9%	72.7%	786.3%[3]	0.00%
	AS_Q5	500	2019	90.6%	86.6%	934.7%[3]	0.00%
Mote Site C	C2	500	2019	98.2%	93.2%	1073.3%[3]	0.00%
	C3	500	2019	98.6%	92.8%	925.1%[3]	0.00%
	C4	500	2019	99.0%	93.6%	473.3%[3]	0.00%
Looe Key	LK_W1	550	2018	68.4%	50.9%	506.2%[3]	1.82%
	LK_W2	800	2019	94.3%	90.3%	1029.5%[3]	0.00%
Summerland Ledges	IC_U_2	250	2020	93.2%	84.8%	-	0.00%
	IC_U_3	250	2021	93.2%	88.8%	-	0.00%
Cook Island	CI_NFHspawn	870	2011–2017	-	-	-	-
Cat's Paw	CP_M1	345	2019	89.9%	78.0%	N/A	51.30%
	CP_M3	220	2020	99.5%	95.9%	N/A	94.09%
Dog's Leg	DL_M1	500	2020	92.8%	74.4%	N/A	70.00%
	DL_M3	315	2021	99.7%	95.5%	N/A	40.95%

Table S2. Detailed description of workflow and settings for processing Structure-from-Motion (SfM) data using the software Agisoft Metashape v2.0.

Step	Procedure	Description	Settings
1	Acquisition	Diver-based images (Johnson et al. 2025) were captured at a swim height of 1–2 m from the reef base using a double-lawnmower swim pattern ensuring 70–80% overlap between swim passes. Cameras were set to shoot in RAW format, continuous shoot mode. Total area covered by diver-based imagery was ~5300 m^2.	Cameras used:<br>Canon EOS R (surface) - site IC-U2 only<br>Dual Nikon D7000 18 mm and 55 m lenses (Mote Marine Lab) - site IC-U1 and IC-U3<br>Canon Powershot S120 (at 2 m above the reef) - all other sites
2	Image pre-check	Images were downloaded from camera SD card in RAW format and checked to ensure images were "landscape" orientation. Images were then converted to non-lossy TIFF format using the batch processor in Adobe Photoshop ®. Image files were saved with 'date_site_transect_imagenumber' filename and placed in folders by site and transect for import into Agisoft Metashape.	Open Photoshop→File→Scripts→Image Processor→Select Folder containing images to process and location to save processed images. Check Save as TIFF. Check LZW Compression>Click Run
3	Import images	Images were batch loaded into Agisoft Metashape and processed using an Ace x64-based PC with the following configuration: Intel ® Xeon ® Gold 6334 CPU @3.60GHz, with 8 Cores,16 logical processors, and 128GB RAM.	Open a new workspace in Metashape→Click Workflow→Add Folder→Navigate to the site folder that contains imagery→Select Folder→Single Camera→Create chunk from each subfolder
4	Image check	EXIF data were checked to ensure accuracy. Image quality was estimated and images with image quality <0.50 were disabled. Images from the different collections were placed into separate camera groups.	In Metashape right click any image in the Photos Pane>Estimate Image Quality→Entire Workspace.<br><br>To place images in groups: Select images in Photo Pane→Right click→Move Images→New Image Group

5	Alignment	Images were aligned in a local coordinate system (m) to create a sparse point cloud.	Workflow→Batch Process→Add→Job type: Align Photos→Apply to: Select chunks<br>Accuracy: High<br>Generic preselection: Yes<br>Key point limit: 60000<br>Tie point limit: 0<br>Exclude stationary tie points: yes<br>All other options: no
6	Optimize camera alignment	After initial alignment, a least squares bundle adjustment was applied to each transect to refine the alignment and solve for better camera calibration. Used default parameter settings.	Workflow→Batch Process→Add→Job type: Optimize Alignment→Apply to: Select chunks>Use defaults
7	Add markers and scale	Each transect was scaled using 3–4 25 cm coded scalebar targets placed throughout the sampling area.	Tools→Markers→Detect Markers→Circular 12-bit→Tolerance: start with 20. If all coded markers were not detected, increase the tolerance.
8	Error reduction	A custom script was used to reduce the number of low-quality points and improve model accuracy. "Reconstruction Uncertainty" threshold was between 20–40. "Projection Accuracy" threshold was between 3–4. "Reprojection Errors" set to 0.3. After error reduction, "Optimize Cameras" tool was used one final time with fit additional corrections checked. RMSE values before error reduction ranged from 0.55–2.22 and after ranged between 0.27 and 0.52, an average percent decrease in error of 65%. Maximum horizontal accuracy of 3.41 mm	Download the automated error reduction script here:<br>https://doi.org/10.5066/P9DGS5B9 (Logan et al. 2022)<br>In Metashape, Tools→Run Script→Locate script and add arguments.

9	Image color correction (optional)	Underwater images usually have strong color and contrast modifications from light absorption and scattering. A batch color correction script was applied to transect images to improve final data product visualization. The original raw images were replaced with color-corrected images before building data products. This replacement was conducted by resetting each image path from the raw image to the color-corrected image. Note: image filenames must be identical.	Download the color correction script 'OrthoImage_Color_Correction_Procedure.m' located in the orthoimagery metadata information of the USGS data release located here: http://doi.org/10.5066/P93RIIG9. (Hatcher et al. 2022)<br><br>Open the script in Matlab or Octave change the path in and path out locations. Click→Run
10	Image dehaze (optional)	A final step to reduce haze and shadows in some images was performed in Adobe Photoshop. This was achieved by creating a custom action with a Camera Raw filter that adjusts the tonal width and intensity of shadows, midtones, and highlights.	Camera Raw filter parameters were set as follows: Exposure +0.10, Contrast +35, Highlights -50, Shadows +50.
11	Model orientation and positioning	Models were placed in a user-defined local coordinate system using the zero-point center 1m cell-size grid and the USGS Metashape Alignment Helper Tool.	Download the Alignment Helper tool (https://doi.org/10.5066/P9YN4KDX; Jenkins and Kupfner Johnson 2024) and watch the video walkthrough with instructions for use in the READ ME file.
12	Build point cloud	Three-dimensional color corrected dense point clouds were generated. Note: Bounding box was set to the area of interest.	Right click any photo→Change Path→Choose path to enhanced images. Filenames must be identical to the RAW images.<br><br>Workflow→Batch Process→Add→Job type: Build Point Cloud→Apply to: Select chunks<br>Quality: High<br>Depth filtering: Mild<br>Calculate point colors: yes<br>Estimate tie point covariance: yes

13	Segmentation and filters	Dense point clouds were segmented into three classes which were used to filter data: Low-point noise, canopy, and reef base. Low-point noise includes point with a confidence <2. Canopy includes all objects with vertical tree-like structure such as gorgonians and branching sponges. Outplants includes staghorn coral outplants. Reef base includes stony corals, substrate, encrusters, and barrel sponges.	Step 1. Create noise filter: Tools→Point Cloud→Filter by confidence→Enter min 0, max 1→Locate the Free-Form Selection tool in the selection tool drop down>lasso all points with confidence →Right click→Assign class→Select Low-point(noise)<br><br>Step2. Create additional filters. Tools→Point Cloud→Reset Filter→Tools→Point Cloud→Select Filter→uncheck Low Point Noise→Free-form Selection→lasso features of interest in point cloud→Right click→Assign→select a feature class. For this project we used "medium vegetation" for canopy, "ground" for reef base, "man-made object" for outplants, "low-point noise" for noise.
14	Build surface elevation models (DSMs)	Digital surface models (x,y,z raster of elevation values) were generated from the segmented point clouds. One DEM per segmentation filter was generated. Bounding box was set to remove edge noise and only include the 10x1m area of interest prior to building DSMs.	Workflow→Batch Process→Add→Job type: Build DEM→Apply to: Select chunks<br>Source data: Point cloud<br>Quality: High<br>Point classes: Select point classes<br>Interpolation: Enable<br>All other settings keep as default
15	Build orthomosaics	Two-dimensional color-corrected orthomosaic images were generated.	Workflow→Batch Process→Add→Job type: Build Orthomosaic>Apply to: Select chunks<br>Surface: DEM<br>Blending mode: Mosaic<br>Check Enable hole filling
16	Export SfM data products	All SfM products were batch exported using the USGS Metashape Export Helper tool.	Download the Export Helper tool (https://doi.org/10.5066/P1C7KKAP; Jenkins and Johnson 2025) and watch the video walkthrough with instructions for use in the README file.

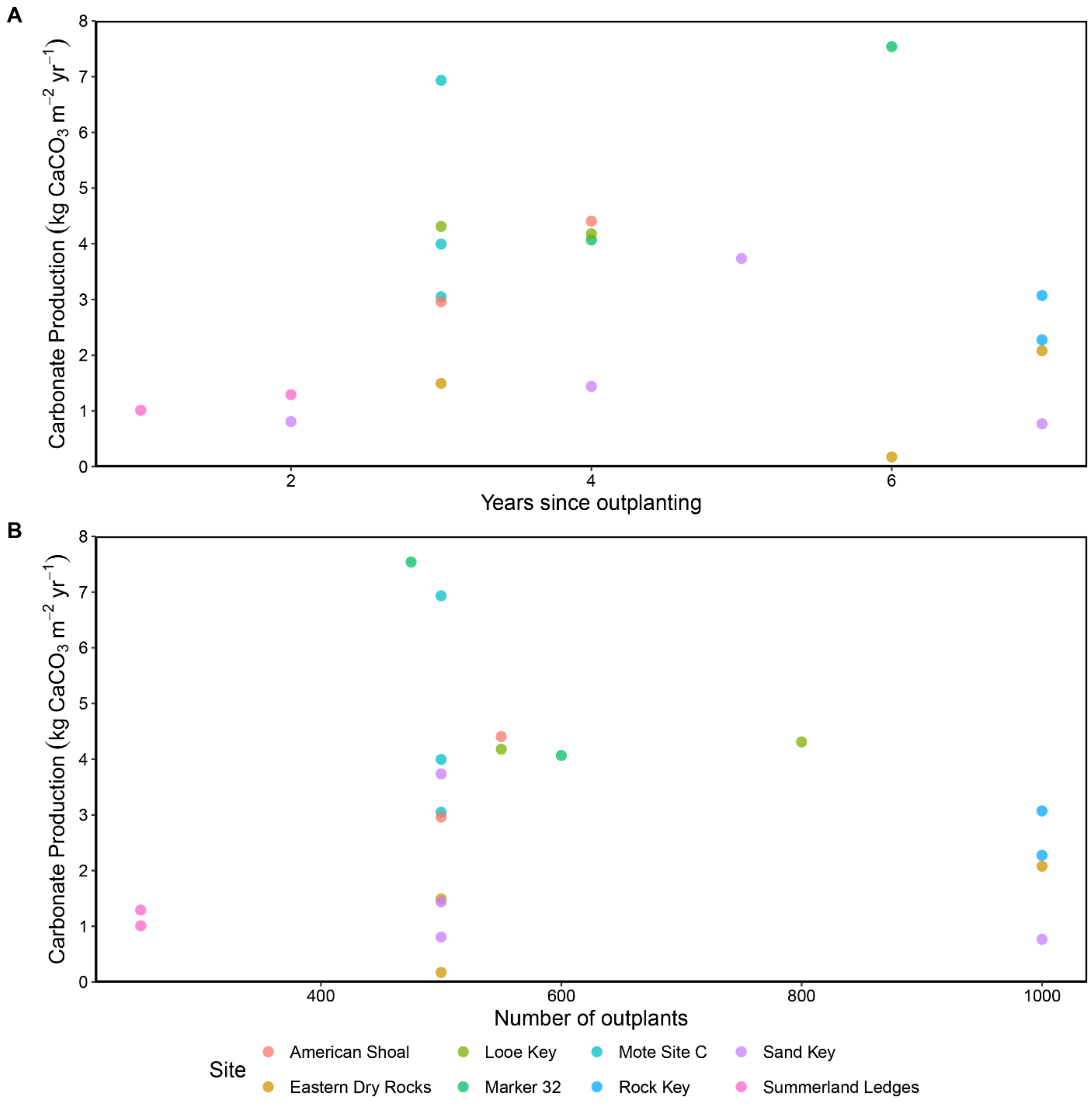

Figure S1. Plots of A) time since outplanting (in years) and B) number of outplants versus gross carbonate production at offshore subsites (points).

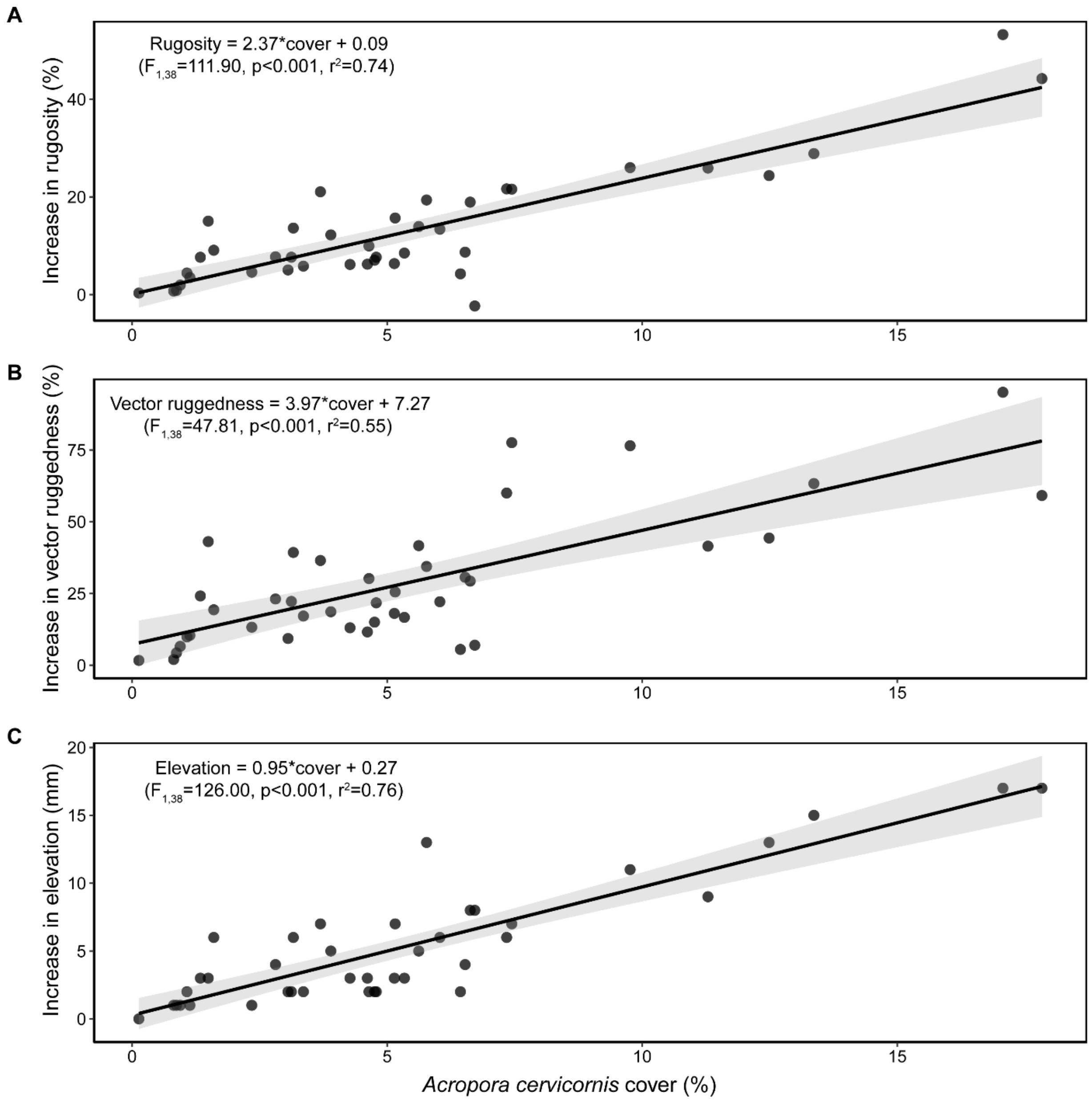

Figure S2. Linear relationship (black line; shading represents one standard error) between outplanted *Acropora cervicornis* cover (points) and increases in A) rugosity, B) vector ruggedness, and C) elevation.

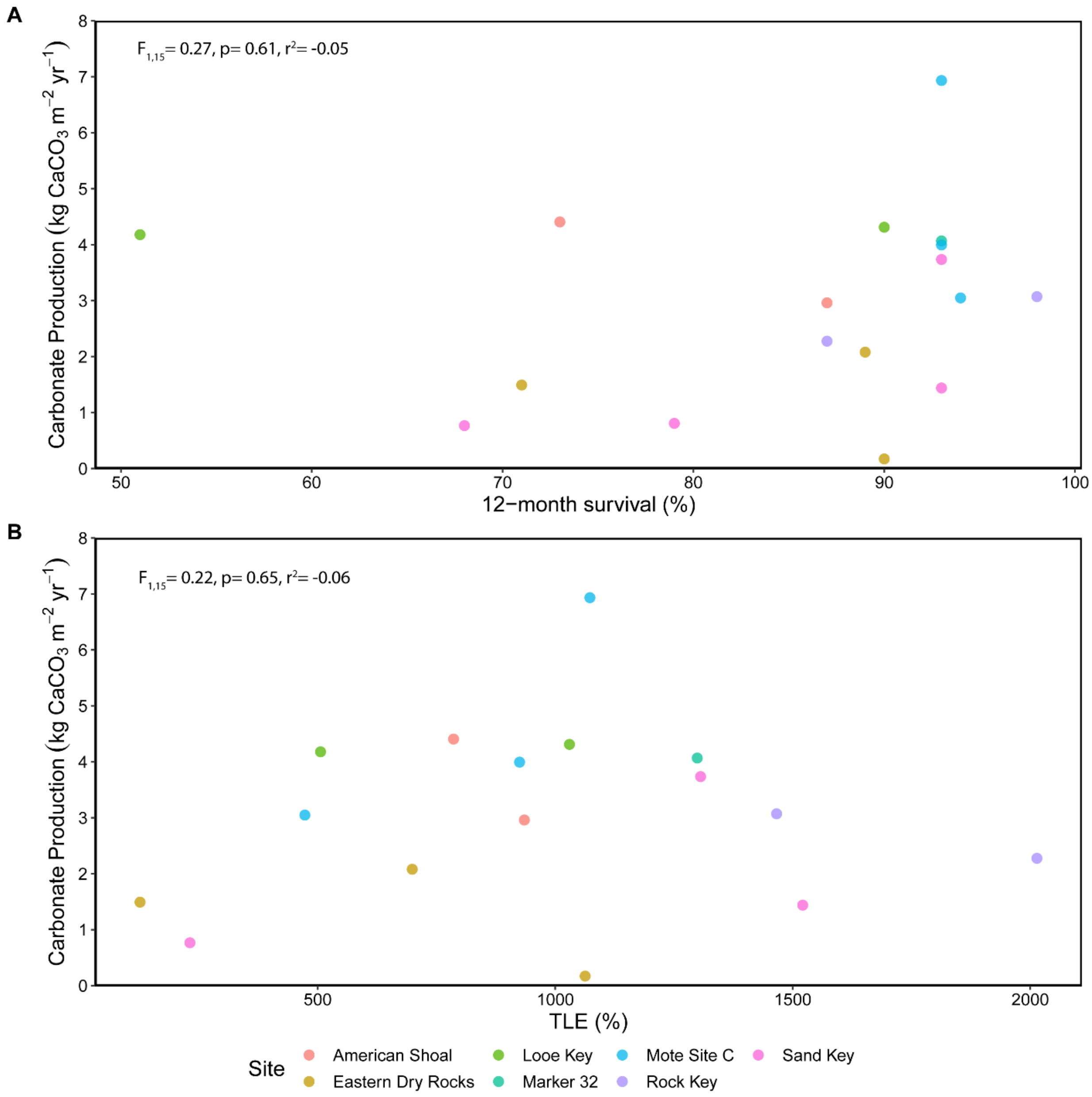

Figure S3. Plots of A) outplant survival after 12 months and B) percent (%) increase in total linear extent (TLE) of outplants 3–5 years after outplanting versus gross carbonate production at offshore subsites.

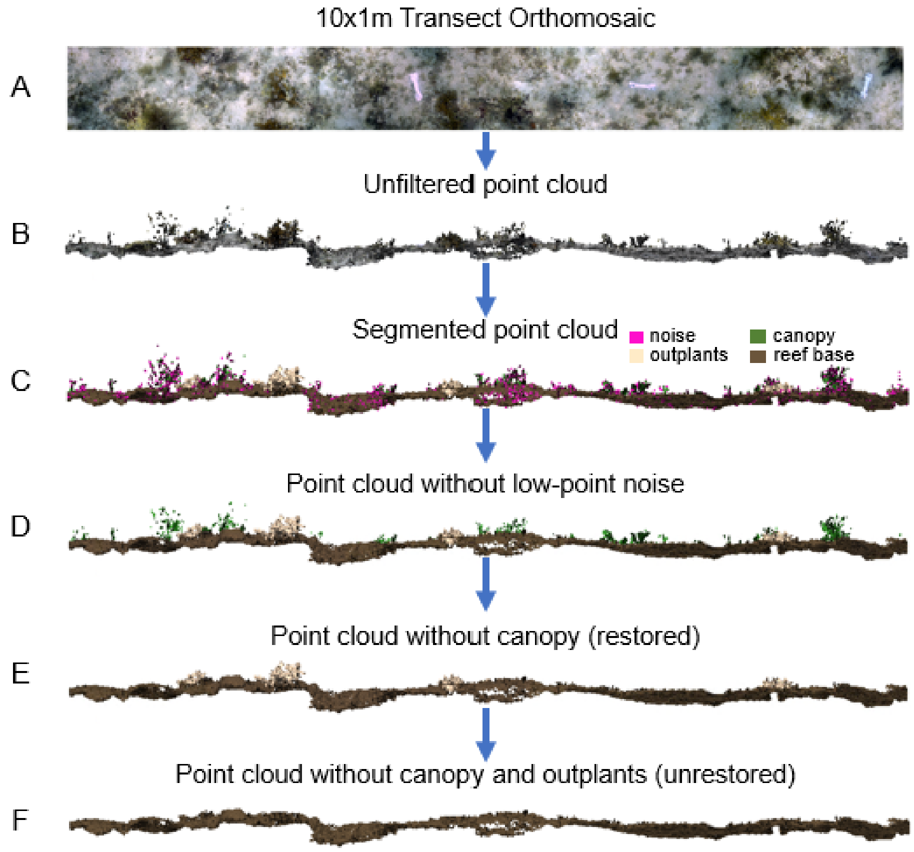

Figure S4. Example of the Structure-from-Motion point cloud segmentation and filtering process: A) transect orthomosaic, B) unfiltered point cloud, C) unfiltered point cloud after segmentation, D) confidence filtered point cloud, E) canopy filtered point cloud, and F) outplant and canopy filtered point cloud.

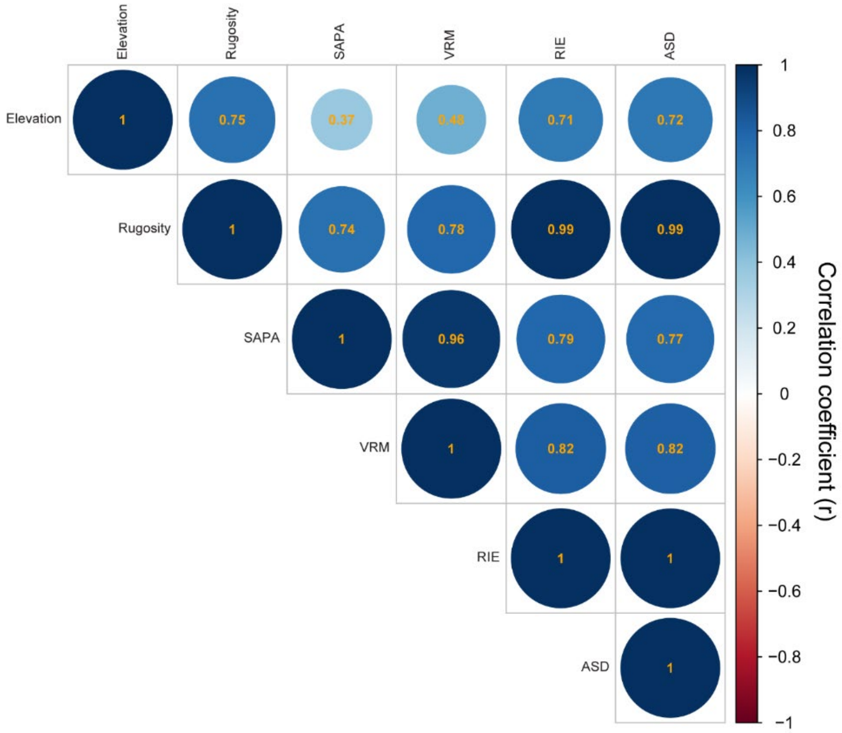

Figure S5. Correlation plot of mean reef structural complexity metrics evaluated for the Structure-from-Motion (SfM) models, calculated as described in (Toth et al. 2025). Elevation is the mean reef elevation standardized to the minimum elevation of the transect in meters. Rugosity is the ratio of the three-dimensional (3D) surface area to the two-dimensional (2D) planar surface area of the SfM models. SAPA, VRM, RIE, and ASD, are calculated as averages across 5 x 5-cm focal windows within the SfM models with values range from zero (smooth) to infinity (rough). SAPA (surface area to planar area ratio) is the mean of the arc-chord corrected (Du Preez 2015) surface area to planar area ratio. VRM (vector ruggedness) is the dispersion of unit vectors normal to the terrain surface. RIE (roughness index of elevation) is the local standard deviation of the residual topography surface. ASD (adjusted standard deviation) is the local standard deviation after removing the influence of slope. Pearson correlation coefficients were obtained using RStudio *corrplot* package. Positive correlations are displayed in blue and negative correlations in red. Color intensity and size of the circle are proportional to the correlation coefficients. The legend on the right side of the correlogram shows correlation coefficients and corresponding colors.

Literature Cited

Du Preez, C. A new arc–chord ratio (ACR) rugosity index for quantifying three-dimensional landscape structural complexity. *Landscape Ecology* **30**, 181–192. https://doi.org/10.1007/s1098 0-014-0118-8 (2015).

Hatcher, G. A., Kranenbug, C. J., Warrick, J. A., Bosse, S. T., Zawada, D. G., Yates, K. K., & Johnson, S. A. Overlapping seabed images and location data acquired using the SQUID-5 system at Eastern Dry Rocks coral reef, Florida, in May 2021, with derived point cloud, digital elevation model and orthomosaic of submerged topography. *U.S. Geological Survey data release*. http://doi.org/10.5066/P93RIIG9 (2022).

Jenkins, C. M., & Kupfner Johnson, S. A. Agisoft Metashape Alignment Helper Version 1.0: *U.S. Geological Survey software release*. https://doi.org/10.5066/P9YN4KDX (2024).

Jenkins, C.M. & Johnson, S.A. Agisoft Metashape Export Helper Version 1.0. *U.S. Geological Survey software release*. https://doi.org/10.5066/P1C7KKAP (2025).

Johnson, S. A., Toth, L. T., Jenkins, C. M. & Lyons, E. A. Diver-based Structure-from-Motion imagery from coral reef restoration surveys in the lower Florida Keys: July 2022 and July 2023. *U.S. Geological Survey data release*. https://doi.org/10.5066/P1WHKTRD (2025).

Logan, J. B., Wernette, P. A. & Ritchie, A. C. Agisoft Metashape/Photoscan Automated Image Alignment and Error Reduction version 2.0: U.S. Geological Survey code repository. *U.S. Geological Survey software release*. python package, Reston, VA. https://doi.org/10.5066/P9DGS5B9 (2022).

Toth, L. T., Johnson, S. A., Lyons, E., Jenkins, C. M., Stathakopoulos, A., Bloomer, S. K., Mallon, J., & Combs, I. Carbonate budgets, Structure-from-Motion products, and topographic complexity measurements from restored and non-restored areas of coral reefs in the Lower Florida Keys. *U.S. Geological Survey data release*. https://doi.org/10.5066/P13HMEON (2025).
